# Piezoelectric Actuated Phase Shifter Based on External Laser Interferometer: Design, Control and Experimental Validation

**DOI:** 10.3390/s17040838

**Published:** 2017-04-11

**Authors:** Peng-Zhi Li, Xiao-Dong Wang, Yong-Xin Sui, De-Fu Zhang, Dong-Fang Wang, Li-Jian Dong, Ming-Yang Ni

**Affiliations:** 1 Changchun Institute of Optics, Fine Mechanics and Physics, Chinese Academy of Sciences, Changchun 130033, China; suiyx@sklao.ac.cn (Y.-X.S.); zhangdf@sklao.ac.cn (D.-F.Z.); donglj@ciomp.ac.cn (L.-J.D.); nimy@sklao.ac.cn (M.-Y.N.); 2 University of Chinese Academy of Sciences, Beijing 100049, China; wangdf@sklao.ac.cn

**Keywords:** piezoelectric actuators, flexure, semi-closed loop, laser interferometer

## Abstract

To improve the phase-shifting accuracy, this paper presents a novel integrated framework for design, control and experimental validation of the piezoelectric actuated phase shifter with a trade-off between accuracy and cost. The piezoelectric actuators with built-in sensors are adopted to drive the double parallel four-bar linkage flexure hinge-based mechanisms. Three mechanisms form the tripod structure of the assembled phase shifter. Then, a semi-closed loop controller with inner feedback and outer feedforward loops via the external laser interferometer is developed for accurate positioning of the phase shifter. Finally, experiments related with travel range, step response, linearity and repeatability are carried out. The linearity error is 0.21% and the repeatability error of 10 μm displacement is 3 nm. The results clearly demonstrate the good performance of the developed phase shifter and the feasibility of the proposed integrated framework.

## 1. Introduction

Phase-shifting interferometry [1,2,3,4,5] is generally used for the interferometric optical testing, especially in Fizeau interferometers. The phase difference is made between two interfering beams and the object phase can be retrieved from a series of interferograms using a suitable algorithm. Mounting the reference mirror on a piezoelectric actuated phase shifter is most prevalent when altering the phase difference between the two interfering optical beams.

Discovered in 1880, the piezoelectric effect is commonly known as the electromechanical interaction between the mechanical strain/force and the electrical field/charge in some solid crystalline materials. It is a reversible process. The lead zirconate titanate crystals will change about 0.1% of their static length when an electric field is applied because of the inverse piezoelectric effect. They are used in the production of the piezoelectric actuators. Piezoelectric actuators are widely used in micro-/nano-positioning [6,7,8,9,10], micro-/nano-manipulation [11,12], atomic force microscope [13,14], vibration suppression [15] and optics [16,17,18] due to high bandwidth and nanometer displacement resolution.

Piezoelectric actuated phase shifter provides a practically efficient way of improving the phase-shifting accuracy. Although there are commercial products available in the market, the accuracy and cost of the phase shifter can hardly be properly balanced. A highly accurate phase shifter is often operated in closed loop control with the displacement feedback of costly capacitive sensors. On the other hand, a low-cost phase shifter is generally characterized by open loop control with poor linearity. Hence, this paper presents a novel integrated framework for design, control and experimental validation of the phase shifter with a trade-off between accuracy and cost. The piezoelectric actuators with built-in sensors are adopted to drive the flexure hinge-based mechanisms. The double parallel four-bar linkage flexure mechanisms are designed and analysed to cause Z/Tip/Tilt motion of the phase shifter by forming the tripod structure [19,20]. Then, a semi-closed loop control strategy is developed for accurate positioning of the phase shifter. The inner loop control is implemented with the feedback of built-in sensors in the piezoelectric actuators. The outer loop control is realized with the feedforward of the cubic polynomial linearization and motion transmission matrix via the external ultra-precise laser interferometer. Experiments concerning travel range, step response, linearity and repeatability are carried out to validate the performance of the designed phase shifter.

The rest of this paper is organized as follows. Section 2 presents the structure design of the phase shifter. The phase shifter is assembled with three piezoelectric actuated flexure mechanisms. In Section 3, the control architecture is introduced based on the inner closed-loop control and outer open-loop control. In Section 4, experiments are investigated to check the performance of the phase shifter. Section 5 concludes the whole paper.

## 2. Structure Design

### 2.1. Piezoelectric Actuator

The stacked piezoelectric actuator is chosen for driving the phase shifter. It is mechanically sealed outside for better protection of inner multi-layered piezoelectric ceramic stacks. The preloaded actuator is produced by Harbin Core Tomorrow Science and Technology Co., Ltd. in Harbin, China. There is a built-in resistance strain gauge sensor (SGS) in the actuator to monitor its displacement. In addition, the actuator is customized with a ball tip, which is useful for decoupling torque and off-center forces. The nominal travel range is 16 μm. More specifications are listed in Table 1.

It should be noted that SGS is applied mainly for cost-cutting reasons. The SGS is composed of a resistive film bonded to the piezoelectric stacks. When strain occurs, the film resistance changes correspondingly. Generally, four or more strain gauges form a Wheatstone electric bridge driven by a direct current voltage. When the bridge resistance changes, the SGS signal conditioner converts the resulting voltage change into a signal proportional to the displacement.

### 2.2. Flexure Hinge-Based Mechanism

**Figure 1:**
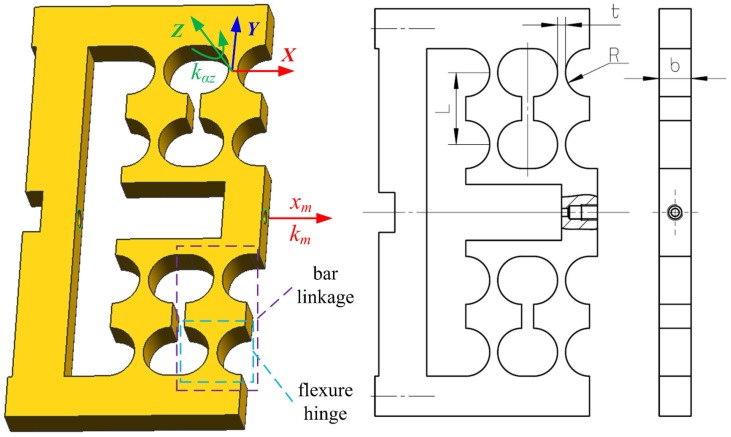
Double parallel four-bar linkage mechanism.

Concerning the motion transmission and guiding mechanism, right-circular flexure hinges are adopted. The flexure hinge is characterized by non-friction and non-stiction based on the elastic deformation of a solid material. Shown in Figure 1, a double parallel four-bar linkage mechanism is designed to avoid planar coupling motion in other directions than the applied force. Two flexure hinges form one-bar linkage, and two-bar linkages make up one parallel linkage mechanism, and then two parallel linkages finally constitute the designed double parallel linkage mechanism. Therefore, the mechanism is composed of eight right-circular flexure hinges.

The material type of the flexure hinges is 45 steel and its physical characteristics is shown in Table 2.

When the piezoelectric actuator operates against the flexure hinge-based mechanism, the available displacement is related to the stiffness of the mechanism by the following equation [21]:

(1) $$x_{m}\approx x_{a}\frac{k_{a}}{k_{a}+k_{m}},$$

where $x_{m}$ is the displacement of the mechanism, $x_{a}$ is the displacement of the piezoelectric actuator under the exciting voltage when operated without external load, and $k_{m}$ and $k_{a}$ are the stiffness of the mechanism and actuator, respectively. In order to keep down the loss of the displacement range, the stiffness of the mechanism is usually designed to be under 1/10 that of the actuator. As a result, the mechanism without lever amplification will generate approximately the same maximum displacement as the nominal displacement of the actuator.

The angular stiffness $k_{\alpha z}$ of a single right-circular flexure hinges on the *z*-axis of the local coordinate system can be computed in a simplified equation [22]:

(2) $$k_{\alpha z}=\frac{2Ebt^{\frac{5}{2}}}{9\pi R^{\frac{1}{2}}},$$

where *E* is the elastic modulus of the mechanism material, and *b*, *t* and *R* are width, thickness and radius of a single right-circular flexure hinge in Figure 1, respectively.

The stiffness of the mechanism in the direction of the actuator elongation can be approximated by the equation [23] based on the potential energy analysis method as follows:

(3) $$k_{m}=\frac{8k_{\alpha z}}{L^{2}},$$

where *L* is the length of the bar linkage in Figure 1. Substituting $k_{\alpha z}$ into Equation (2) into the equation above yields

(4) $$k_{m}=\frac{16Ebt^{\frac{5}{2}}}{9\pi L^{2}R^{\frac{1}{2}}}.$$

The dimensions of the mechanism are listed in Table 3.

**Figure 2:**
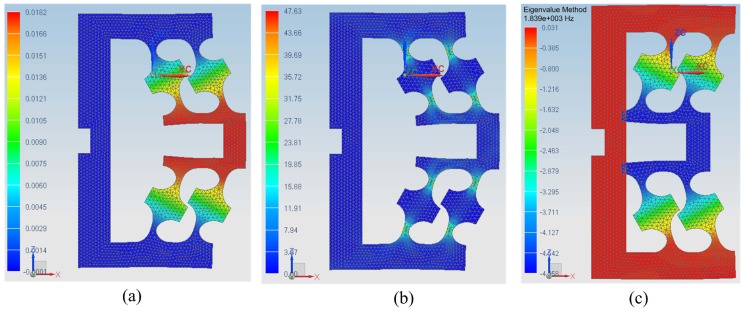
FEA results of the mechanism: (**a**) maximum displacement is 0.0182 mm; (**b**) maximum Von Mises stress is 47.63 MPa; (**c**) natural frequency in the actuated direction is 1839 Hz.

Using the values in Table 1, Table 2 and Table 3, according to Equations (1) and (4), we have $k_{m}=6.78N/\mu m<k_{a}/10=70N/\mu m/10=7N/\mu m$ and $x_{m}\approx 14.6\;\mu m$. Finite element analysis (FEA) is used to compute the displacement and natural frequency of the mechanism without load and further check the stress intensity based on an NX V8.0.0.25 NASTRAN solver [24,25] of Siemens AG in Berlin, Germany, shown in Figure 2. The maximum Von Mises stress 47.63 MPa of the mechanism in Figure 2b is lower than the allowable stress 355 MPa in Table 2. Figure 2c shows that the natural frequency of the mechanism without load in the actuated direction is 1839 Hz, which implies fast dynamical response of the developed mechanism.

### 2.3. Phase Shifter

**Figure 3:**
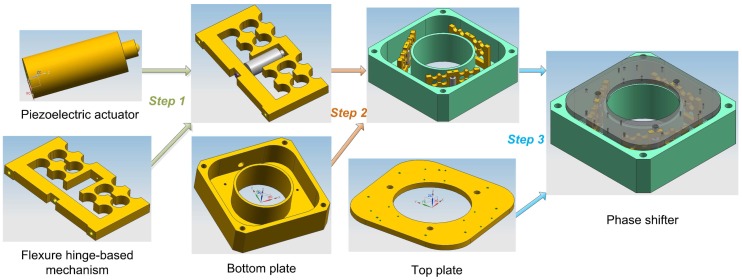
Assembly procedures of the phase shifter.

The phase shifter is assembled with three piezoelectric actuators, three flexure hinge-based mechanisms, one bottom plate, one top plate and some screws. As shown in Figure 3, the assembly procedures can be executed step by step as follows:

- Step 1: Assemble the actuator with the mechanism. Firstly, make the ball tip of the actuator closely contact with the loading end of the mechanism; then, push tightly the bottom of the actuator with a slotted set screw through the thread hole of the fixing end of the mechanism.
- Step 2: Connect the assembly finished in Step 1 together with the bottom plate via six screws with identical torque.
- Step 3: Connect the assembly finished in Step 2 together with the top plate by three screws with identical torque.

Finally, we have completed the assembly of the phase shifter.

Hence, three piezoelectric actuated flexure mechanisms form a tripod structure of the phase shifter. Because of manufacture and assembly tolerance, the upper surface plane of the top plate is not completely parallel to that of the bottom plate. In other words, the heights of three legs of the tripod are not identical. The height variation, i.e., the parallelism between the upper surface plane of the top plate and that of the bottom plate can be measured by coordinate measuring machine (CMM). Then, by mechanical grinding, the washers of different thickness can be obtained. Using the adjusting washers between the actuated mechanism and the top plate, suitable parallelism within acceptable tolerance can be achieved.

**Figure 4:**
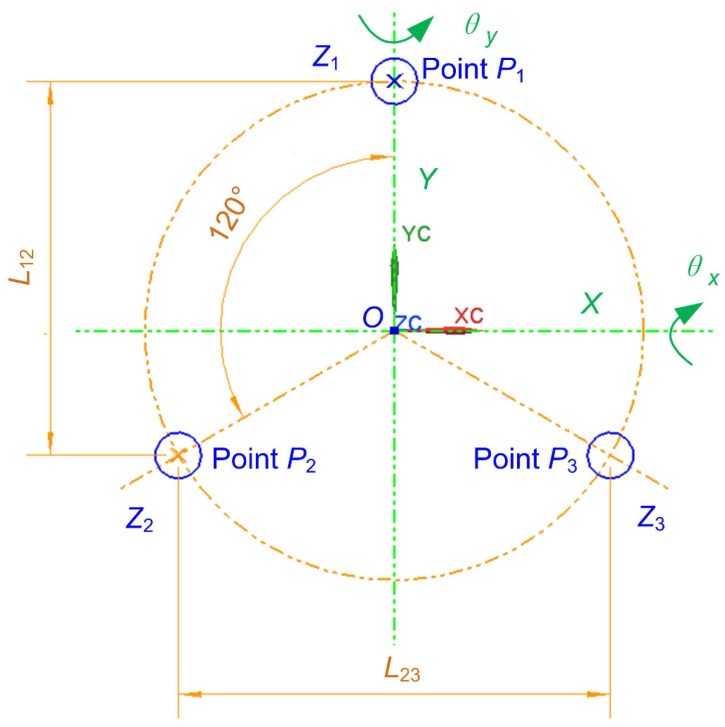
Schematic diagram of the tripod structure of the phase shifter.

As the moving part of the phase shifter, the top plate is viewed as a rigid body, which means that the distance between any of its two points remains constant and its deformation is neglected. Illustrated in Figure 4, the relationship between the displacement of the phase shifter with a tripod structure and that of the three different points of the top plate is described in the following equation [20]:

(5) $$\begin{array}{cc} Z & =\frac{Z_{1}+Z_{2}+Z_{3}}{3},\\ \theta_{x} & =\frac{2Z_{1}-Z_{2}-Z_{3}}{2L_{12}},\\ \theta_{y} & =\frac{Z_{2}-Z_{3}}{L_{23}}. \end{array}$$

In Equation (5), *Z* represents the linear displacement of the phase shifter on the *z*-axis, $\theta_{x}$ and $\theta_{y}$ are the angular displacement of the phase shifter on the *x*-axis and *y*-axis, respectively, $Z_{1}$, $Z_{2}$ and $Z_{3}$ are the linear displacement of the three points $P_{1}$, $P_{2}$ and $P_{3}$ of the top plate on the *z*-axis, respectively. *Z* is usually described by the linear displacement of the mass centroid, i.e., the origin *O*. $Z_{1}$, $Z_{2}$ and $Z_{3}$ are generally measured using the displacement sensors. In the ideal condition, if the three actuated linkage mechanisms have the same displacement and thus result in the equal displacement $Z_{1}$, $Z_{2}$ and $Z_{3}$ of the three points $P_{1}$, $P_{2}$ and $P_{3}$, the phase shifter will only move in the direction of the *z*-axis with $\theta_{x}=\theta_{y}=0$.

## 3. Control Architecture

**Figure 5:**
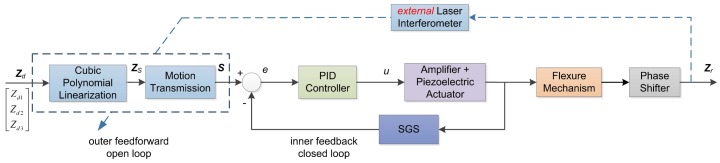
Diagram of the semi-closed loop controller.

The semi-closed loop control strategy is employed for a trade-off between accuracy and cost. As shown in Figure 5, there are two control loops: inner closed loop for feedback control and outer open loop for feedforward control. The inner loop uses discrete-time incremental PID control algorithm [23,26] in Equation (6) to guarantee accurate displacement of the piezoelectric actuator. The feedback displacement is supplied by built-in SGS. The outer loop adopts cubic polynomial linearization in Equation (7) and Jacobi motion transmission matrix in Equation (8) to compensate for the nonlinear relationship of displacement between the actuator and phase shifter.

(6) $$\begin{array}{cc} u(k) & =u(k-1)+\Delta u(k),\\ \Delta u(k) & =k_{p}\left(e(k)-e(k-1)\right)+k_{i}e\left(k\right)+k_{d}\left(e(k)-2e(k-1)+e(k-2)\right), \end{array}$$

(7) $$Z_{S}=p_{3}{Z_{d}}^{3}+p_{2}{Z_{d}}^{2}+p_{1}Z_{d}+p_{0},$$

(8) $$S=\left[\begin{array}{c} S_{1}\\ S_{2}\\ S_{3} \end{array}\right]=AZ_{S}=\left[\begin{array}{ccc} A_{11} & A_{12} & A_{13}\\ A_{21} & A_{22} & A_{23}\\ A_{31} & A_{32} & A_{33} \end{array}\right]\left[\begin{array}{c} Z_{S1}\\ Z_{S2}\\ Z_{S3} \end{array}\right].$$

In Equations (6)–(8), $k_{p},k_{i}$ and $k_{d}$ are the proportional gain, integral gain and derivative gain, respectively, $Z_{S}$ represents the cubic polynomial linearized displacement of the phase shifter, $Z_{d}$ and S are the desired displacements of phase shifter and piezoelectric actuators, respectively, $p_{3},p_{2},p_{1}$ and $p_{0}$ are coefficients of the cubic polynomial, and A is the motion transmission matrix from phase shifter to piezoelectric actuators. As shown in Figure 5, $Z_{d1}$, $Z_{d2}$ and $Z_{d3}$ of the input $Z_{d}$ of the whole controller are the desired displacements of the three points, $P_{1}$, $P_{2}$ and $P_{3}$, in Figure 4. They are often expected to be identical, i.e., $Z_{d1}=Z_{d2}=Z_{d3}$, in order to make the phase shifter move only in the direction of the *z*-axis. Under such conditions, the desired $Z=Z_{d1}$ and $\theta_{x}=\theta_{y}=0$. The output $Z_{r}$ of the whole controller are the real displacements of the three points $P_{1}$, $P_{2}$ and $P_{3}$ in Figure 4, which in this paper are measured by the external laser interferometer.

The parameters of the designed controller above are determined from the inner loop to the outer loop in the following sequence:

- First, $k_{p},k_{i}$ and $k_{d}$ of PID controller are chosen through the trial and error method [26]. Gradually increase the values of $k_{p},k_{i}$ and $k_{d}$, and then check the dynamic characteristics of the step response of the piezoelectric actuator until little overshoot, short settling time and small stable error are obtained. To guarantee the stability of PID controller, step response to the maximum nominal displacement is stably adjusted with little or even no overshoot. In this way, sufficient stability margin can be achieved.
- Second, the motion transmission matrix A from phase shifter to piezoelectric actuators is determined with normal work of PID controller. Move the three piezoelectric actuators individually for a certain displacement in closed loop control, measure and record the corresponding displacements of the three points $P_{1}$, $P_{2}$ and $P_{3}$ of the phase shifter, respectively, and then compute A based on the displacement data of the actuators and phase shifter in the least square method. This procedure could be repeated many times to attain average values of A.
- Third, with normal work of both PID controller and the motion transmission matrix, the coefficients $p_{3},p_{2},p_{1}$ and $p_{0}$ can be obtained by fitting the relationship between the desired displacement and the real one of the phase shifter using the cubic polynomial. During the travel range, input 10 or more desired displacements with equal intervals to the working controller, measure and record the corresponding output displacements of the phase shifter, and then linearize the input and output displacement data with a cubic polynomial.

The overall control algorithm is computationally simple and can be easily implemented in a low-cost chip microprocessor. In addition, no expensive sensors like capacitive sensors and optical grating encoders are needed other than the built-in SGS.

The feedforward control needs an external laser interferometer to supply more accurate displacement measurement. The measured data are used to determine the parameters of the polynomials and transmission matrix. It should be noted that the laser interferometer works offline, i.e., the normal work of the designed controller won’t involve the laser interferometer.

**Figure 6:**
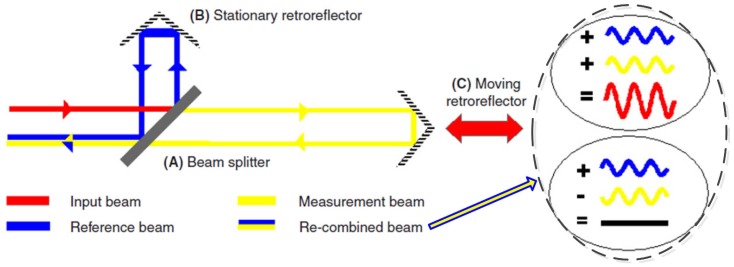
Working principle of the laser interferometer.

The laser interferometer can provide ultra-precise linear displacement measurement. The working principle of the laser interferometer is illustrated in Figure 6. The light wavelength emerging from the laser is usually 633 nm. The light from the laser is split into two beams by beamsplitter (A), and then about half the laser light is sent to the stationary retroreflector (B) and forms the reference beam. The other half strikes the moving retroreflector (C) and forms the measurement arm. The retroreflectors return the two beams back to the beamsplitter where they recombine and interfere with each other. If the measurement beam path length is changed (retroreflector C moves), the relative phases of the interfering beams will alter. The resulting cycle of constructive and destructive interference will cause the cyclic variation of the intensity in the recombined beam. Displacement is thus measured by counting these cycles. The measuring uncertainty are mainly from system errors, environmental errors and geometric errors. The laser interferometer generally consists of a laser kit, a compensator kit, a linear measurement kit and software.

## 4. Experiments

**Figure 7:**
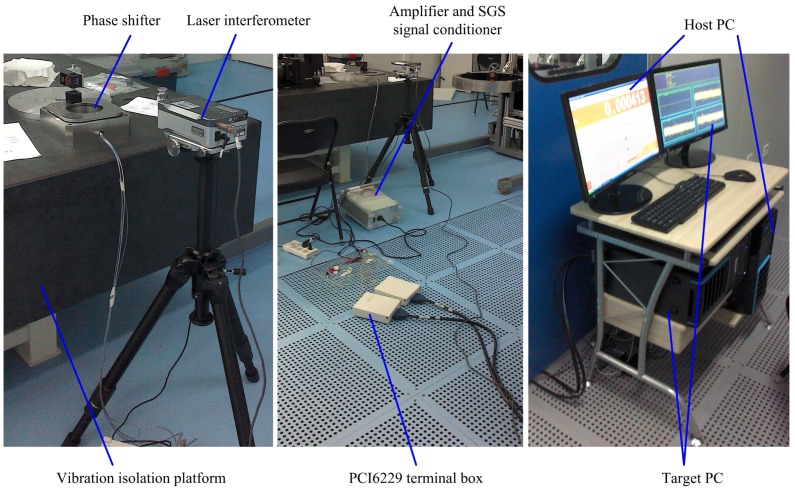
The experimental equipment.

**Figure 8:**
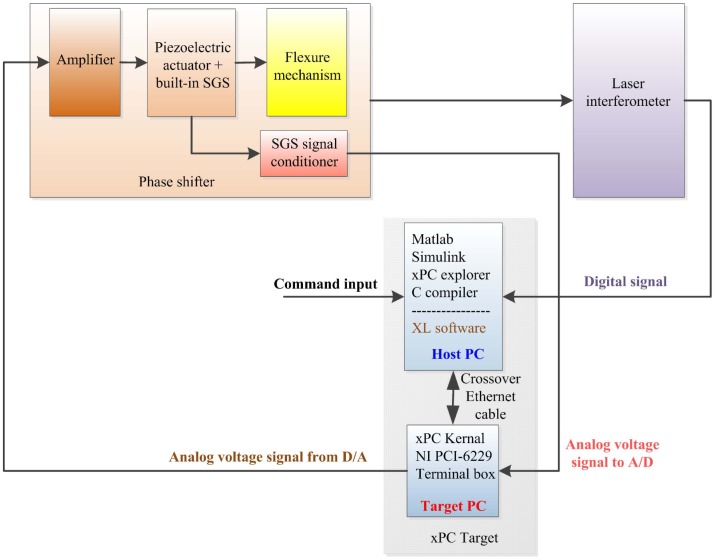
The experimental schematic diagram.

The experiments are carried out in a laboratory under precise environmental control. The temperature of the laboratory is kept at 22 ± 0.06 Celsius. The laboratory is also constructed as a class 1000 clear room. The environment of this kind gives a sufficient guarantee for ultra-precise displacement measurement. The experimental equipment is shown in Figure 7, which mainly consists of three parts: (1) phase shifter, (2) laser interferometer and (3) xPC Target™ 4.2 of MathWorks, Inc. in Natick, MA, USA. The phase shifter is placed on an optical vibration isolation platform for rejecting external vibrational disturbance. Illustrated in Figure 8, the experimental schematic diagram shows the main components , the signal flow and data transfer. The three parts and their relevant components of the experimental equipment could be introduced as follows:

- (1) Phase shifter is assembled using the piezoelectric actuators and flexure mechanisms. The piezoelectric actuators are excited by power amplifiers with 15× gain and monitored by built-in SGS displacement sensors. The amplifier can amplify the input 0–10 V analog voltage by 15 times and output 0–150 V analog voltage with an average power of 7 W. The SGS signal conditioner converts the signal generated from SGS into 0–10 V analog voltage with a 0.1% nonlinearity. The flexure mechanisms are manufactured by wire cutting electrical discharge machine.
- (2) The Renishaw XL-80 system manufactured in Gloucestershire, UK is employed as the laser interferometer. Its resolution can reach 1 nm and system accuracy can be up to 0.5 ppm. The measured data can be displayed, updated and logged by XL software in host PC. The linear measurement optical kit is fixed on the top surface of the phase shifter to measure the linear vertical displacement.
- (3) xPC Target can run Simulink${}^{®}$ models on a target PC for rapid control prototyping with a library of I/O device drivers and a real-time kernel. The hardware consists of a host PC, a target PC, I/O boards in the target PC, and a network connection between the host and target computers. The software is made up of MATLAB${}^{®}$, Simulink, xPC explorer, a C compiler and xPC real-time kernel. It can convert the Simulink model created in the host PC into the target application running on the target PC in real time. In the experiments, the I/O data acquisition board is PCI-6229 device from National Instruments (NI) Corporation in Austin, TX, USA. The NI PCI-6229 board has 16 differential analog input channels and four analog output channels. Its ADC and DAC resolutions are both 16 bits. The connection between the host PC and target PC is realized via a crossover Ethernet cable.

The working processes of the signal flow and data transfer of the experiments can be depicted in the following way:

- When a command input comes, xPC Target runs the semi-closed loop control application based on SGS measurement and outputs analog voltages to the amplifiers.
- The SGS measurement is generated from the SGS signal conditioner and then connected to the analog input channels of NI PCI-6229 board through the terminal box.
- The analog voltage output of xPC Target is accomplished by means of the analog output channels of NI PCI-6229 board.
- The amplifiers drive the piezoelectric actuators based on their input analog voltages. Then, the piezoelectric actuators elongate and result in the corresponding displacements of the flexure mechanisms.
- Meanwhile, the generated displacement of the phase shifter is measured by the laser interferometer and then transferred to XL software in the host PC for data displaying and logging.

**Figure 9:**
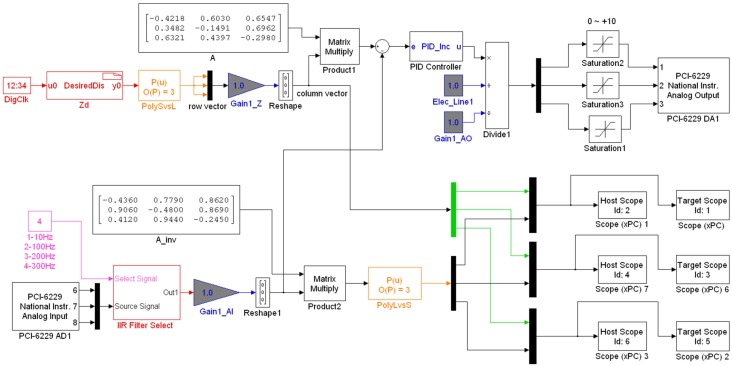
The Simulink model for the real-time semi-closed loop control target application.

The original Simulink model for the real-time semi-closed loop control target application is shown in Figure 9. The blocks *PolySvsL*, *A* and *PID Controller* are used for cubic polynomial linearization, motion transmission matrix and PID controller, respectively. Scope blocks are utilized for data displaying and recording. Other blocks such as *PCI-6229 AD1* and *PCI-6229 DA1* in the xPC Target library provide real-time interfaces to the NI PCI-6229 board, through which the Simulink model can collect the analog voltage signal from SGS and send the analog voltage signal to the amplifier. The sampling time $T_{s}$ is 1 ms and other parameters of the controller are as follows:

$$\begin{array}{cc} k_{p} & =0.6,k_{i}=0.2,k_{d}=0.001,\\ p_{3} & =-0.0001009,p_{2}=0.000389,p_{1}=1.014,p_{0}=0.2914,\\ A & =\left[\begin{array}{ccc} A_{11} & A_{12} & A_{13}\\ A_{21} & A_{22} & A_{23}\\ A_{31} & A_{32} & A_{33} \end{array}\right]=\left[\begin{array}{ccc} -0.4218 & 0.6030 & 0.6547\\ 0.3482 & -0.1491 & 0.6962\\ 0.6321 & 0.4397 & -0.2980 \end{array}\right]. \end{array}$$

### 4.1. Travel Range and Step Response

**Figure 10:**
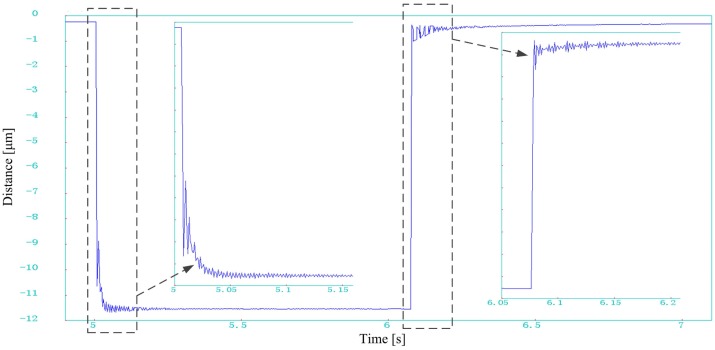
The step response to a desired 11 μm displacement of the phase shifter. Data are captured by the external laser interferometer.

Under the open loop control, the phase shifter is excited by the maximum voltage of 150 V and the corresponding maximum displacement is 12.9 μm (0.012931 mm) measured by the laser interferometer. When operated in the semi-closed loop control, the phase shifter can reach a maximum displacement of 11.5 μm. Figure 10 shows the dynamic response of the phase shifter to a desired step. The step displacement is 11 μm. The data are captured in the dynamic measurement mode of the laser interferometer at 1 kHz sampling rate. It can be seen that the phase shifter can make 11 μm step movement accurately in less than 0.3 s under semi-closed loop control.

### 4.2. Linearity

**Figure 11:**
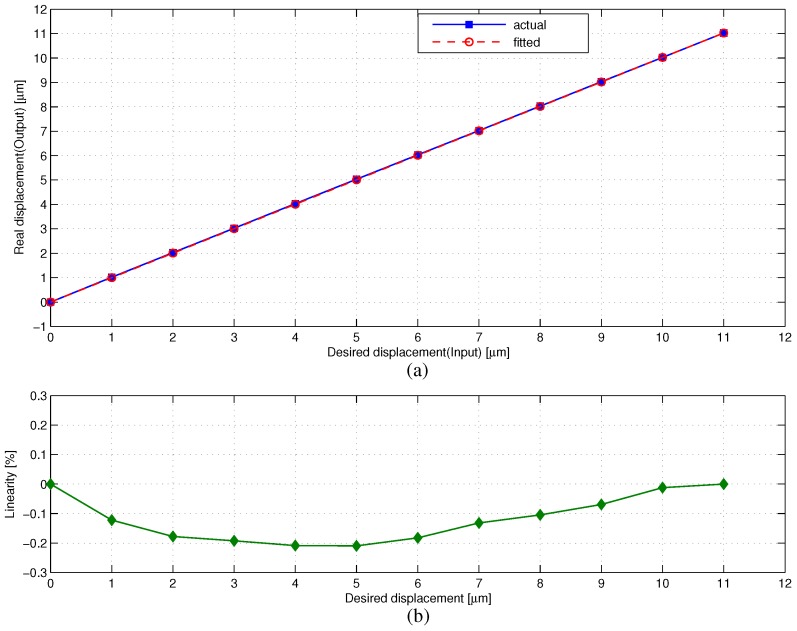
Linearity of the phase shifter: (**a**) displacement curve; (**b**) linearity error.

Linearity means the error between the real displacement and the first-order polynomial fitted displacement. The linearity test of the phase shifter starts from zero point to the displacement of 11 μm in semi-closed loop control. The movement increment is 1 μm and lasts 10 s. The data are captured in the linear measurement mode of the laser interferometer with 1 s sampling interval. As illustrated in Figure 11, the linearity error is 0.21% based on the zero-end point linear fitting method.

### 4.3. Repeatability

**Figure 12:**
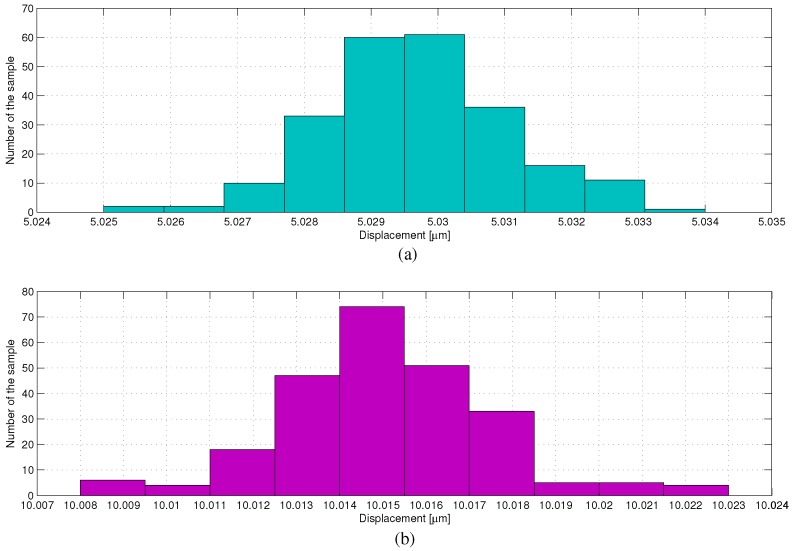
Repeatability of the phase shifter: (**a**) displacement distribution at 5 μm ($e_{R}$ = 2 nm); (**b**) displacement distribution at 10 μm ($e_{R}$ = 3 nm).

Repeatability means the error when the same displacement occurs many times. The repeatability tests of the phase shifter are carried out under the conditions that the phase shifter moves 5 and 10 μm from zero point separately. The repeatability error $e_{R}$ is calculated in Equation (9) as follows [27]:

(9) $$\begin{array}{cc} e_{R} & ={\left(\frac{1}{n-1}\sum_{i=1}^{n}{\left(z_{i}-\bar{z}\right)}^{2}\right)}^{\frac{1}{2}},\\ \bar{z} & =\frac{1}{n}\sum_{i=1}^{n}z_{i}, \end{array}$$

where *n* denotes the number of the total sampled displacement and $z_{i}$ represents the *i*th sampled displacement of the phase shifter. Figure 12 shows the displacement distribution when the phase shifter moves 5 and 10 μm individually for 230 times. The repeatability errors at the displacements of 5 and 10 μm are 2 and 3 nm, respectively.

At last, the experimental results of the phase shifter are summarized in Table 4. For comparison, Table 4 also includes the technical specifications of some similar commercial products. It should be noted that no available standard products can be directly used as the phase shifter without modification or customization. S315.10 [28] from Physik Instrumente in Karlsruhe, Germany is operated in an open loop without any sensor. By asking the technical supporting engineer, we know that the linearity error is poor above 2%. Integrated with costly capacitive sensors, NPS-Z-15H [29] from Queensgate in London, UK can keep the linearity error down to 0.02%. Consequently, the phase shifter developed in this paper has a performance of good accuracy with moderate cost.

## 5. Conclusions

In this paper, the piezoelectric actuators with built-in sensors are adopted to drive the right-circular flexure hinge-based mechanisms with a trade-off between accuracy and cost. The double parallel four-bar linkage flexure mechanisms are designed to form the tripod structure of the assembled phase shifter. Then, a semi-closed loop control strategy is developed for accurate positioning of the phase shifter. The inner loop PID control is implemented with the feedback of built-in sensors in the piezoelectric actuators. The outer loop control is realized with the feedforward of the cubic polynomial linearization and motion transmission matrix based on the measured data of the external ultra-precise laser interferometer. At last, experiments with regard to travel range, step response, linearity and repeatability are carried out. The travel range is more than 10 μm, the linearity error is 0.21% and the repeatability error is within the range of 3 nm. The experimental results show that this paper presents a feasible integrated framework for developing a piezoelectric actuated phase shifter with good performance at proper cost.

## Figures and Tables

**Table 1:** Technical specifications of the adopted piezoelectric actuator.

Stiffness	Pushing Force	Pulling Force	Capacitance	Natural Frequency	Length
70 N/μm	1400 N	150 N	1.8 μF	30 kHz	28 mm

**Table 2:** Physical characteristics of the material of the flexure hinge.

Elastic Modulus	Poisson Ratio	Density	Allowable Stress
2.1 × 10${}^{11}$ Pa	0.28	7800 kg/m${}^{3}$	355 MPa

**Table 3:** Dimensions of the flexure hinge and bar linkage.

*b*	*t*	*R*	*L*
8 mm	2 mm	6 mm	18 mm

**Table 4:** Experimental results of the phase shifter.

	Travel Range	Step Response	Linearity	Repeatability (1σ)
This paper	12.9 μm@open loop	<0.3 s @11 μm	0.21%	2 nm @5 μm
	11.5 μm@semi-closed loop			3 nm @10 μm
S315.10	12 μm@open loop	-	2%–4%	-
NPS-Z-15H	15 μm@closed loop	-	0.02%	-

## Abbreviations

The following abbreviations are used in this manuscript:

SGS	Strain gauge sensor
FEA	Finite element analysis
CMM	Coordinate measuring machine
PID	Proportional, integral and derivative
NI	National instruments
ADC	Analog-to-digital converter
DAC	Digital-to-analog converter
